# Supplementary figures and images for: Forecasting drug resistant HIV protease evolution
Source: PLoS Comput Biol. 2026 Jan 27;22(1):e1013913. doi: 10.1371/journal.pcbi.1013913 (PMC12858072; doi:10.1371/journal.pcbi.1013913)

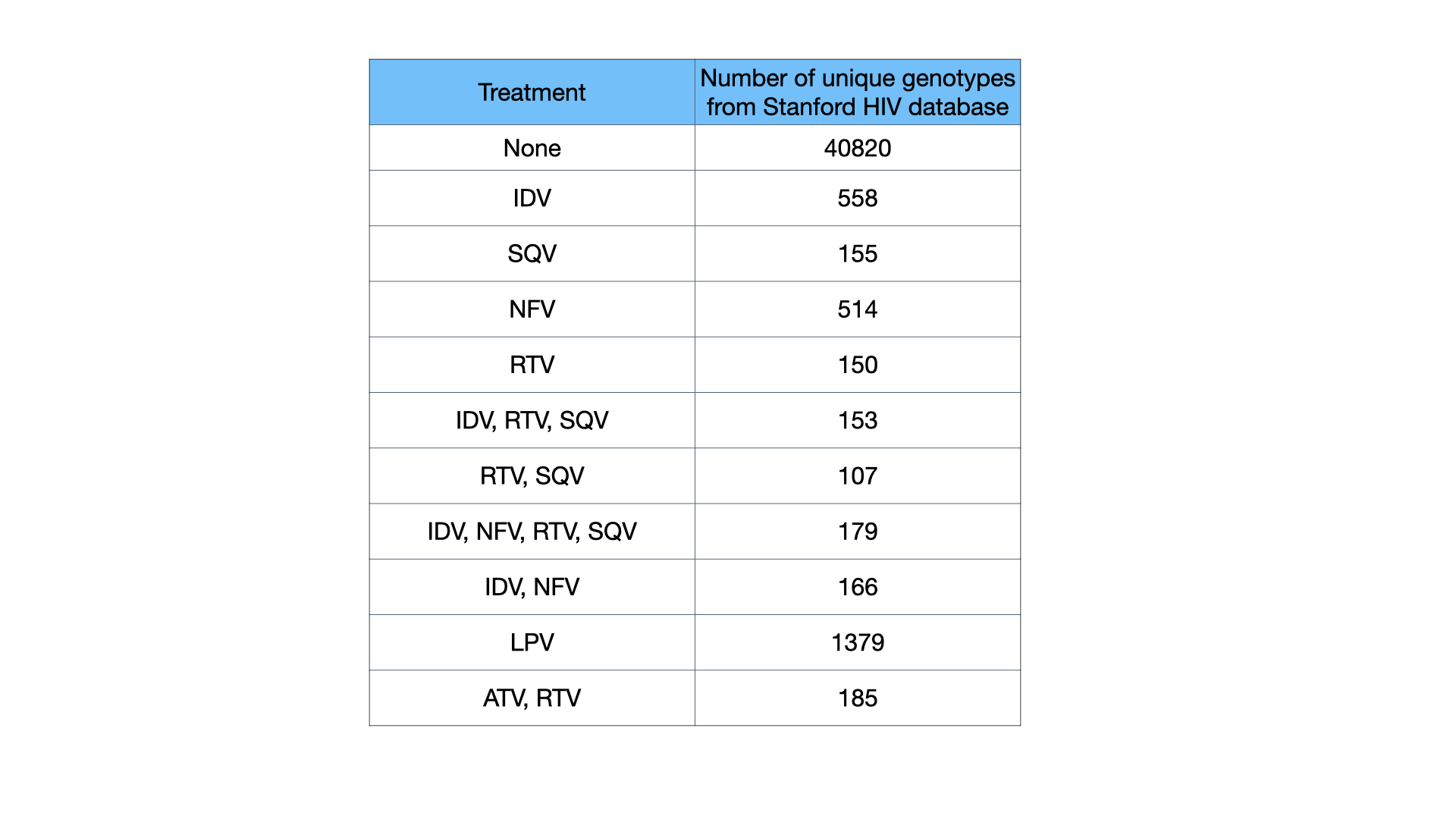

Supplement: S1 Table — (TIFF) [file pcbi.1013913.s001.tiff]

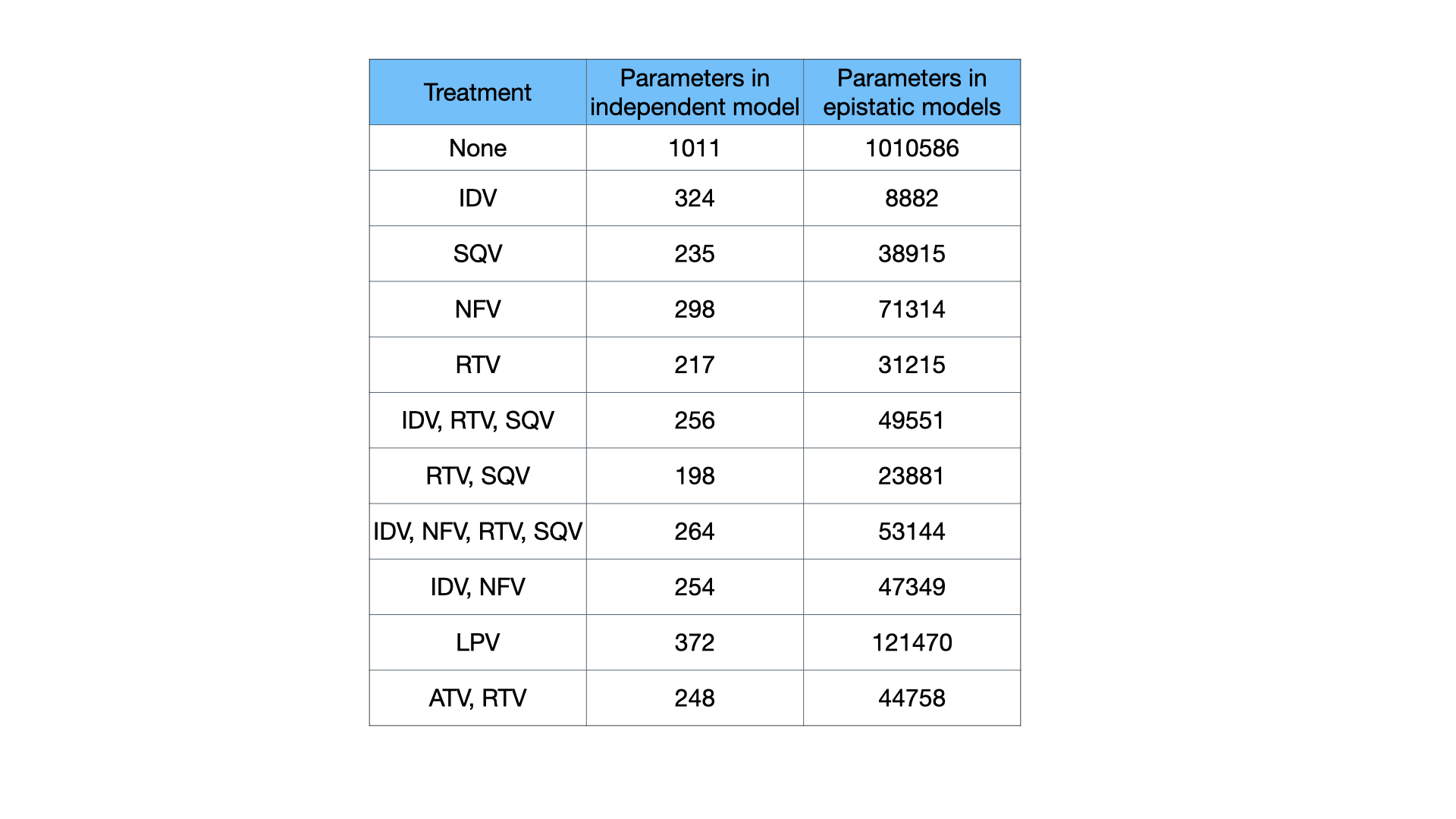

Supplement: S2 Table — (TIFF) [file pcbi.1013913.s002.tiff]

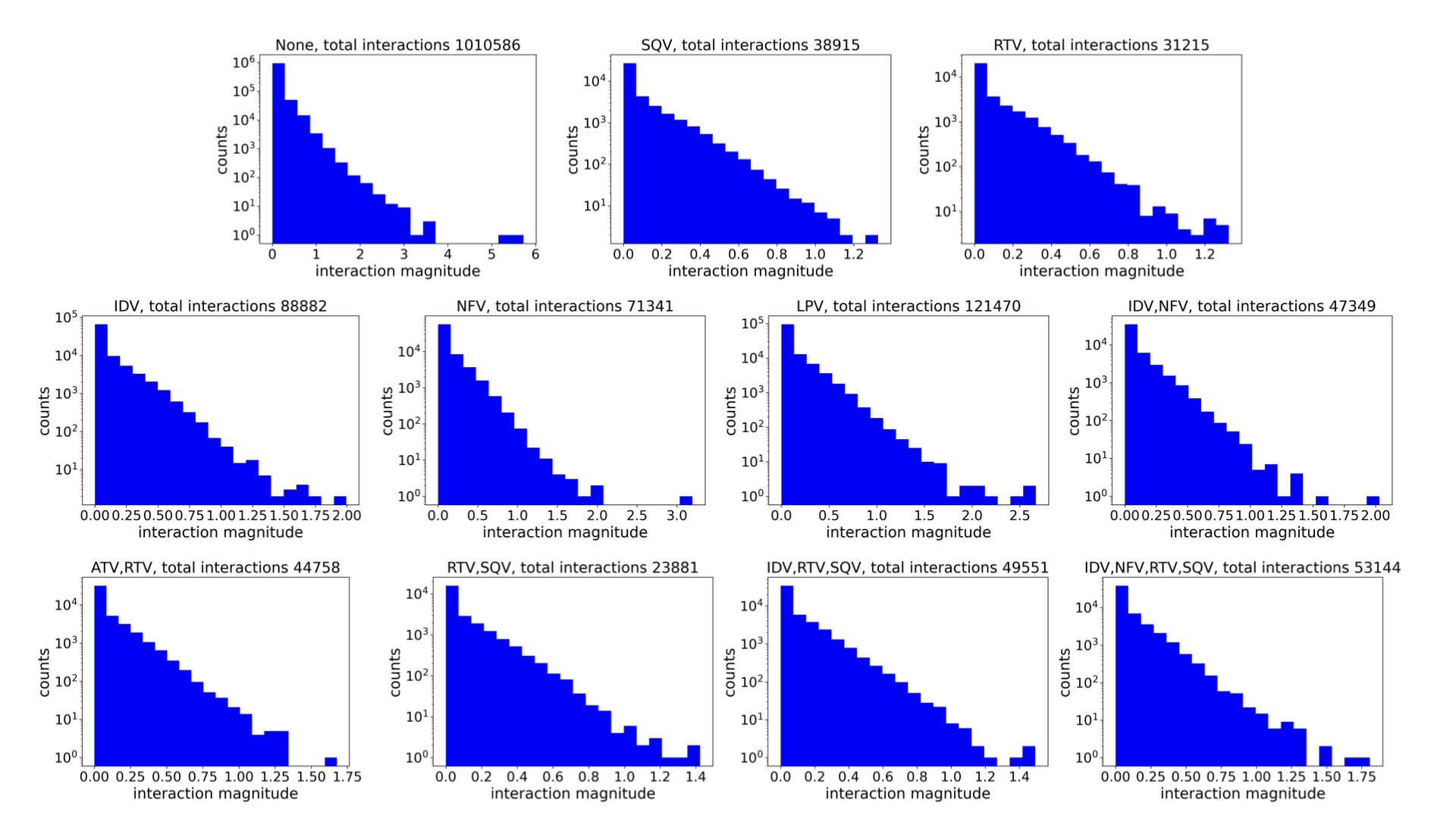

Supplement: S1 Fig — (TIFF) [file pcbi.1013913.s003.tiff]
