# Supplementary material for: Forecasting drug resistant HIV protease evolution
Source: PLoS Comput Biol. 2026 Jan 27;22(1):e1013913. doi: 10.1371/journal.pcbi.1013913 (PMC12858072; doi:10.1371/journal.pcbi.1013913)
Supplement: Attachment — Submitted filename: Response_to_reviewers_PLOS_HIV.docx [file pcbi.1013913.s004.docx]

Reviewer #1: The paper considers profiles of mutations in the protease enzyme in drug-naive and drug-treated HIV samples. It builds a probabilistic profile of the different amino acids that appear at all loci throughout the protein in different data subsets, and uses this profile to build a model for the "mutation propensity" of a given genotype under a given drug treatment. This mutation propensity is both interrogated itself, intepreted as linked to fitness, and also used to simulate putative evolutionary trajectories under different circumstances. A combination of these pictures is used to build a picture of genotypes that are both evolutionarily accessible and high fitness (under the promises of the model). Finally, a collection of particular mutations that are predicted to challenge the emergence of accessible, fit genotypes are identified.

This is an interesting paper and the appearance of previously-identified mutations of importance in the final analysis provide some support for its validity. There's also an interesting identification of a known, but less understood, mutation as a particularly important "driver" of drug resistance evolution. There is interesting science here. But it is, at the moment, obfuscated by a combination of unintuitive writing, confusing methods, and a collection of results that distract from (rather than support) the main scientific picture. Fixing these would both focus on the key ideas and make them more interpretable.

A collection of comments are below, starting with some cross-cutting general ones and then some more specific points. For transparency, I am Iain Johnston and I am happy for this review to be treated as public domain. To my eyes, my most important shortcomings as a reviewer here are a lack of familiarity with the specific "FEM" inference method central to the paper, and a lack of experience with the molecular biology of HIV protease.

Top-level comments

1. The most important variables throughout the manuscript are called things like
"negative log likelihood to not mutate" / "FEM-fitness"
"log(-log prob to reach)"
and the collection of double negatives involved really challenges intuitive intepretation. I strongly suggest the authors introduce shorthand for these quantities (perhaps things like "mutation propensity" and "accessibility" respectively) to help at-a-glance interpretation of the text and figures. I particularly think "FEM fitness" should be avoided, as it pictures fitness as forward-looking (propensity for future mutation). I don't think any of the scientific story suffers if "[predicted] mutation propensity" is used instead.

We now call “negative log likelihood to not mutate“ the **mutation propensity** and "log(-log prob to reach)" the **1/reachability**.

We have replaced FEM with logistic regression models, and we have removed ‘FEM fitness’.

2. Section 2.2, and other parts to a lesser extent, present results which are largely or wholly dependent on a model variable (beta) which I find very hard to tie to a biological observable. It's not clear how much this adds to the scientific story, and in later sections it's not clear what particular choice of this value has been used in evolutionary simulation. Likewise, the large set of PCA visualisations in Fig 1 don't provide much (any?) scientific insight and can readily be removed.

We clarify now that beta is the inverse temperature, and its main purpose is to simulate rare events. We cite relevant literature for this method (Frenkel and Smit, 2001, Earl and Deem, 2005).

We simulated evolutionary trajectories for the same set of different values of beta for all different treatment regimens and leave-one-out cases. Results from all simulations were combined for subsequent analyses. It is important that the same set of beta values is considered for all different cases. We note that the mutation propensities and reachability are computed using P(A_i = alpha | not i) and not (P(A_i = alpha | not i))^beta.

We now provide a possible biological interpretation of beta as inverse of mutability. However, the use of beta is for the computational feasibility of simulating rare events. We give slightly more explanation with relevant citations (Frenkel and Smit, 2001, Earl and Deem, 2005).

We have removed all PCA visualizations.

3. The core technology is free energy minimisation inference, but the positioning of the introduction and the rest of the manuscript with respect to this is very confusing. The technology looks somewhat like a modification of logistic regression, particular as there is no scientifically meaningful "energy". The training approach is never described, and the key quantity P_F is conditioned on a particular point in a potentially vast state space that cannot possibly be spanned by the training set. I may well be misunderstanding, but I can't see how the steps in Eqns. 17-19, which seem very important for the core quantity of interest, follow (see specific comment below). More information about these points is needed -- see individual points below.

We have replaced FEM with logistic regression models for both coevolutionary epistatic training and drug resistance inference.

4. Throughout, the reader is led to wonder if a simpler set of approaches would give comparable results.

What if we used logistic regression instead of FEM inference?

We have done that now.

What if the classification model in Sec 2.3 just contained the core set of amino acid mutations found later as predictors (this analysis should be done)?

We address this at the end of this review (before the review for Methods).

What if "evolutionary accessibility" was just summarised as number of mutations from the reference case? And importantly -- how important actually is epistasis in this system? We are given (unless I'm misunderstanding) no information about how important this conditioning on current genotype actually is for the outputs of the model?

We implemented the independent model in which the probability of observing an amino acid at a position is its frequency in the observed genotypes. Comparison of the epistatic model with the independent model shows that the simulated trajectories have closer proximity (hamming distance from the consensus) to the observed sequence space (new section 2.1). Therefore, we propose to use the epistatic model for subsequent analysis.

More targetted comments

Line numbers would have helped this review!

The manuscript now shows line numbers.

Second para p1 "First". A couple of strange directions. Why is selection deterministic when mutation and drift are stochastic? Fundamentally it is applied by a noisy environment. As a description of evolutionary models this holds; but we are not yet talking about maths, just noisy biology! And -- fitness is generally defined as replicative ability. The determinants of *that* are hugely complicated.

We have re-written it as “A viral evolutionary path is a sequence of evolving protein genotypes that is driven by evolutionary fitness and the stochastic influences of mutations and genetic drift.”

p2
"the effects of a mutation on the properties of a genotype depend on the selective pressures in the environment". This is either true or false depending on what "properties" means, and most reasonable meanings would make it false. The effect of mutation on a genotype don't depend on selection; the effect of mutation on a genotype's later representation in a population do, but via phenotype.

We have re-written it as “the effects of a mutation on the **evolution** of a genotype depend …”

"a specific point-mutation can theoretically have up to 20^L different effects on protein genotypes of length L since there are 20 different amino acids." Not if we're talking about SNPs (most common definition of point mutation), because only a small subset of those 20 amino acids will be accessible via a single polymorphism. Should clarify that you mean point mutation at the protein sequence level.

We have removed this from the introduction for simplicity.

"mutations are randomly selected but weighted by inferred transition probabilities." -- this is very unclear to a reader who is at this point. What does randomly selected mean? What does selected mean if the approach "avoids a mechanistic formulation of natural selection"? What does weighted mean?

We have re-written introduction with the aim to avoid partial information that may be confusing to the reader at this point.

"we do not use an energy model" -- given that you are using free energy minimisation, this is also very confusing at this point and needs more explanation. "forecast the emergence of drug-resistant PR strains that have high FEM-fitness, defined specifically and precisely for our approach in the following" -- but you've previously said that you will avoid a mechanistic formulation of fitness and an energy model. At this point I have no idea what shape the approach is going to have!

We have replaced FEM with logistic regression.

"We define FEM-fitness of a genotype as its likelihood to mutate" -- again confusing, especially as evolution famously cannot look ahead, so fitness cannot possibly be defined by something that will happen in the future. Even if it could, it's not clear what direction this picture works in. Is mutating good (to evolve resistance) or bad (because the vast majority of mutations will be detrimental)?

We have replaced FEM-fitness with mutation propensity.

The use of "log likelihood" is quite confusing here (and throughout). Unless I am misunderstanding, P_F is a probability produced by a trained model, not a likelihood (which is usually used to refer to a probabilistic connection to a particular empirical observation). Why is it a log likelihood and not a log probability? And in fact, as it seems lots of followup will be concerned with comparing drug-treated and drug-naive conditions, why not use a log odds?

We have removed "log likelihood" throughout and call P_F the conditional or transition probability inferred by the trained models.

p3
Effectively all annotation in Fig 1 is too small to read on a reasonable scale. The plotting protocols should be included in the caption (e.g. that 1A-D use PCA).
I can't follow the "negative loglik of genotypes to not mutate" -- there are too many negatives here. The definition of the previous page refers to the relative probabilities of mutation under two different treatment regimes -- it doesn't refer to probabilities of not mutating.

We now call “negative log likelihood to not mutate” the **mutation propensity** and "log(-log prob to reach)" the **1/reachability**.

I half assume that what we are supposed to take from Fig 1 is that the density of points in 1E is above the diagonal, meaning that mutation propensity is systematically higher under drug treatments (vertical axis) than under no treatment (horizontal axis)?

We have replaced the figure with boxplots to directly compare mutation propensities in different treatment regimens and in two different cases of drug-naïve genotypes and treatment-specific observed genotypes.

I cannot see that 1A-D tell us anything about the science? Any structure in PCA space is not mentioned as a scientific message. Unless I am mistaken, they could readily be removed to focus on the important message 1E.

We have removed all PCA plots.

"Briefly" -- we need more information here! Put this in the methods and expand upon it. Especially how beta informs the "importance sampling" -- it's not at all clear that this in fact importance sampling as opposed to a simple weighted sampling of discrete events. What I think is happening is that you are randomly choosing loci to mutate according to some function of their predicted mutation propensity.

We are using the inverse temperature scaling to simulate rare events. We have added citations for this pre-established method.

p4
Fig 2 and Sec 2.2 focus on how the evolutionary simulation depends on beta. But it's not at all clear how this provides a link to the underlying science. What is the real-world observable that beta corresponds to? It seems to me to provide a weighting between a null model where every mutation is equally likely (high temperature) and a more constrained model where mutation probabilities are entirely determined by your fitted model (low temperature). But nature doesn't know about your fitted model, so how should I intepret beta when thinking about the biology?

The main use of beta is to make it computationally feasible to simulate rare events. It is a common approach for stochastic models that is called inverse temperature scaling, and sometimes importance sampling. We now mention it clearly and give citations for this pre-established method.

Consequently, it is important that any comparison uses the same values of beta. We simulate evolutionary trajectories for the exact same set of betas in different treatment regimens, and then we compare the results of all trajectories.

However, we propose a biological interpretation of beta as 1/mutability in the revised manuscript, which is to say that lower beta value implies higher mutability that is constrained less by the trained model.

The section label for 2.3 is misleading -- it's for one particular drug resistance, and the score is rather low for all others. Better to remove the specific value (or at least say the range).

We have renamed the sections and mention the range in the text.

p5
Fig 3B is redundant with and less informative than 3C; remove it. But why is the collection of points at exactly the same y-value (around 14) in 3B not reflected in 3C, and what is its meaning?

The new Figure 5 replaces Figures 3 and 4 in the first submission.

We address the comments though. In the original version, the heatmap also had the points at the same y-value, but it looked different because the density varied widely with the x-value.

We use the scatter plot to illustrate the \phi(\tau) plots at varying values of \tau, which we believe might be helpful to some readers. We do not want to use the 2D heatmap to illustrate \phi(\tau) plots at varying values of \tau because the artificial binning might require clarification that we are not counting bins or coarsening resolution before counting.

What is beta in these simulations?

The results are combined for all different values of beta for which trajectories were simulated. In the revised manuscript, the values of beta are [0.4, 0.5, 0.6, 0.65, 0.7, 0.75, 0.8, 0.9, 1.0]. We mention it now in the manuscript.

For an informative message about the influence of drug treatment here, it would be useful to have plots like 3A and 3C but under the no-treatment regimen, so that we can see the probabilities of NFV resistance emerging without the drug treatment.

We have removed all PCA plots.

We use Figure 6 to show how mutation propensity and reachability (combined into Pareto optimal function phi(tau)) of drug resistant genotypes changes with respect to no-treatment regimen.

p6
The Pareto optimality section directs us to the Methods to learn about what's going on here, but I can't see any information there about it?
I am completely stuck with:
Briefly, we say that a genotype has Pareto optimality φ if the L2 norm of the log likelihood of not mutating and the log log probability of reaching is φ. The features are rescaled to [0, 1] by dividing them by respective upper bounds of 20 and 10. Hence φ ∈ [0, 2].
What I think this is trying to do is combine the "reachability" of a genotype -- I think under the NFV drug treatment, though this isn't clear -- with its probability of being drug resistant.
Without fully understanding this I'm not sure about Fig 5. I think what we're seeing is that treating with lots of drugs at once gives more genotypes that combine this reachability and resistance probability.

Using the updated terminology, φ takes into account the mutation propensity and the reachability. We compute and compare φ only for the drug-resistant genotypes observed in the simulated trajectories in a treatment regimen. We also note that the starting sequence of the analysis is always the consensus B type sequence, which is drug-naïve.

We show in the new Figure 3 that mutation propensity of drug naïve sequences is higher in muti-PI therapy regimens. It is possible that this causes the evolutionary trajectories starting from the drug-naïve consensus B to explore more diverse sequence space. Therefore, we use φ to consider the mutation propensity and reachability with respect to the treatment regimen of the diverse drug resistant genotypes in the diverse sequence space being explored.

p7
It is interesting that this picture pulls out a subset of mutations that are critical for reachability-resistance. If you now take presence/absence of individuals in this set as predictors in another classification model for resistance, does it do better or worse than your approach in Sec 2.3?

We are sorry but this comment was not clear to us. We would be happy to do this analysis if it can be clarified.

The subset of mutations in Figure 7 that we find as critical to reach drug resistance take into account both, evolution simulation and drug resistance inference. For example, we show in the new analysis of epistatic interactions that 1. position 63 is important in epistatic interactions (tables 2 and 3) 2. 63P is not in the top 3 most important predictors (coefficients of logistic regression models trained for drug resistance inference) of resistance to NFV, suggesting that 63P should not affect the NFV resistance classification model on its own. 3. But leaving out 63P significantly changes the φ plot. We believe this shows the complex interplay between evolution and drug resistance.

Methods
--------------

In Eqn. 3 it would help to explicitly define u_j as the cardinality of the alphabet of symbols found at position j (assuming I'm right in this intepretation?).

In Eqn. 8 should a_N1 be a_L1? If not then I am misunderstanding the indexing here and some more explanation would be useful.

In Eqn. 11 should e^omega H on the numerator be e^H? Otherwise it doesn't match Eqn. 21. FEM via Eqn. 11 looks very similar to logistic regression, if sigma_j are considered as binary predictor variables and b an intercept? Can the authors comment on why logistic regression isn't an appropriate formalism for this picture? Also -- free energy minimisation is a much more general process than this specific inference method. Even if that's the name the original authors proposed, it would help to disambiguate this.

I'm worried that Eqns. 17-19 contain some fundamental issues (but I may well be misinterpreting). In going from Eqn. 17 to Eqn. 18 we seem to be saying that a vector can be written as the intersection of a collection of sets, each of which contains a single element of the vector. e.g. (1, 0, 0, 1) = {1} \cap {0} \cap {0} \cap {1}. This can't possibly be what this means but I can't see how else this is meant to be interpreted.

Then in going from Eqn. 18 to Eqn. 19 we seem to be saying that the (joint) probability of a collection of things (whatever the things are, from my previous question) can be written as the product of probabilities of the individual things. But this is only true if the things are independent, and the elements of a onehot vector are certainly not independent -- fixing a 1 means that all the other elements are immediately known. What am I misunderstanding here?

The most important issue is a (complete) omission of the training process. Without this I simply cannot understand how we can learn from this picture. There is a vast space of \tilde{sigma}_\tilde{p} (I'll write this as s_p to avoid pseudo-TeX) vectors, relatively limited training data (a few hundred in some cases, as mentioned in the intro), and it seems very possible that every datapoint might have a unique s_p. Given that the fundamental quantity of interest P_F is conditioned on a particular s_p, it seems very unclear that a collection of training s_p can inform a (disjoint) set of test s_p?

We have replaced FEM with logistic regression and use Python Scikit libraries for both onehot encoding and logistic regression. This significantly simplifies the implementation and we believe it **addresses all issues with methods** mentioned above.

Regarding the limited training data, we have added analysis of epistatic interactions to show that the logistic regressions models have learned some expected patterns. For example, positions 63, 90 etc. are important (section 2.3) and high epistatic interactions correspond to closer spatial proximity of those positions in the PR structure (section 2.2).

However, it is true that this entire framework is completely data-driven.

Reviewer #2: Reviewer Comments
Title: FORECASTING DRUG RESISTANT HIV PROTEASE EVOLUTION
Manuscript id: PCOMPBIOL-D-25-00787
Overall Recommendation: Major Revision
This manuscript presents a probabilistic large-deviation model leveraging Free Energy Minimization (FEM) to simulate HIV protease evolution under selective drug pressure and forecast resistance emergence. While the approach is conceptually innovative and addresses an important biomedical challenge, the manuscript requires major revision due to insufficient methodological clarity, validation gaps, and limited biological interpretation in key sections.
Major Comments:
**1. FEM-Fitness Metric Requires Clearer Justification Across Regimens
The definition of FEM-fitness (Section 2, page 2) as the minimum negative log conditional probability is central to many downstream claims. However, it remains unclear how this metric accounts for differing selective pressures and sampling biases across treatment regimens. Without normalization or cross-regimen calibration, comparisons in Figures 1D–E and Figure 5 may be confounded.**

We compare different treatment regimens by normalizing ‘FEM-fitness’ using the same constants (20 for mutation propensity and 10 for 1/reachability).

2. Lack of Baseline Comparisons for Resistance Prediction Models
The FEM-based resistance classifiers (Table 1) report high F1 scores for some drugs. However, the manuscript lacks comparison between established ML models such as random forests, SVMs, or logistic regression. Without benchmarking, it is difficult to assess whether FEM offers unique advantages over simpler or more interpretable methods.

We have replaced FEM with logistic regression models for both coevolutionary epistatic training and drug resistance inference.

3. Unclear Biological Basis for Intermediate β Values Producing Fit Sequences
The role of β as an inverse temperature parameter to sample rare mutations is reasonable. However, the non-monotonic behavior shown in Figure 2C—where intermediate β values (e.g., 0.6) yield fitter genotypes—requires stronger biological justification or empirical support. This is a key claim and must be substantiated.

The updated results using the logistic regression models, show that the median fitness decreases with increase in beta. Analysis for intermediate beta values at finer resolution (0.6, 0.65, 0.7, 0.75, 0.8) shows how the shape of the distribution changes significantly in this range of beta values.

4. Omission of {ATV, RTV} Combination from Critical Mutation Analysis
The exclusion of the {ATV, RTV} regimen from the critical mutation analysis (Section 2.5, page 6), despite its clinical relevance and interesting resistance profile (Figure 5), seems premature. A deeper exploration—even qualitative—may provide valuable insight into why this combination shows minimal resistance evolution.

We now show the leave-one-out analysis for ATV+RTV in the discussion section. Specifically, we also highlight mutations that are important for reducing drug resistant mutations.

A quick observation is that allowing the consensus residue I at position 54 is important for reduced NFV resistance, and allowing the mutant V at position 54 is important for increased SQV resistance.

This is just a preliminary analysis, and a detailed analysis and comparison of epistatic interactions and drug resistance interactions is required to rigorously determine what makes ATV+RTV special. We believe this to be a future direction of research.

5. Subtype Limitations and Generalizability Not Fully Addressed
All simulations are based on the subtype B consensus protease sequence. Given global subtype diversity, the generalizability of the model’s predictions, especially the identified critical mutations (Figure 6), must be more explicitly discussed in the main text.

This is a crucial point and a direction of future research. We have used the same starting sequence (consensus subtype B) for computational feasibility since the leave-one-out analysis is computationally expensive in the current stochastic framework. Additional analysis is required to investigate effect of different starting genotypes.

We mention this in a new paragraph of discussion now.

Minor Comments:
6. Use standard mutation nomenclature (e.g., "L90M") throughout figures and text for consistency and clarity.

We have edited the legend of Figure 7 to clarify that the leave-one-out analysis specifically means, for example, ‘Leave out mutation 90M’ which is to say that the evolutionary trajectories will never be allowed to have M at position 90 regardless of what the current residue at position 90 is. So, any mutation resulting in M at 90 is not allowed. We considered stating it as X90M, but that might be misleading to some readers by implying that the consensus is X.

We also now mention in the discussion “Finally, we determine that allowing 90M, 10I, 30N, 63P, 54V, 71V, 84V, 84A, and 84C is critical to higher reachability of drug-resistant genotypes with low mutation propensity. With respect to the consensus B type, these correspond to the mutations L90M, L10I, D30N, L63P, I54V, A71V, and I84V/A/C, respectively.”

7. Clarify the interpretation of PCA axes in Figures 1A–B — do they represent meaningful biological separation or are they primarily for visualization?

The PCA plots were only for visualization. We have removed all PCA plots now.

8. Include a reference or explanation for the β-weighted sampling scheme, particularly if inspired by Boltzmann-like approaches.

We have added citations (Frenkel and Smit, 2001, Earl and Deem, 2005) for the method of using inverse temperature to simulate rare events.

Reviewer #3: Eq. 11 defined for binary sequences. However, the authors applied this equation to one-hot encoded protein sequences. One immediate consequence is that the indicator functions for different amino acids at the same positions are no longer independent, as there can be exactly one AA takes the value of 1, every other AAs must be zero. However, in Eq. 19, the authors treat the AAs at a position as if they are independent. I feel this is somewhat unnatural. So i think the authors should address this limitation.

We have replaced FEM with the logistic regression models for coevolutionary epistatic training. However, we still use onehot encoding of the categorical features (residues) at a position to train the logistic regression models that we mention in the revised Methods.

The conditional probability for a particular AA at a position is determined by H, which is a weighted sum of contributions from AAs at other positions. This is similar to the notion of global epistasis in the evolutionary biology literature. Although this types of epistasis can capture epistasis to certain extent, it cannot account for specific interactions between amino acids. I think given the limited amount of data, this is a perfectly legitimate modeling choice. But I also think the authors should discuss the types of epistasis their model can capture. For example, can we expect the model to pick up specific pairwise interactions between residues in contact?

We have now added multiple analyses (sections 2.2 and 2.3) of the coefficients of logistic regression models to show what epistatic interactions have the models learned as important and how they correlate to spatial distance.

Overall, I feel the results section can benefit from more explanation of the method, which may be unfamiliar to many potential readers. So I suggest the authors move some material from the Methods section to the results and provide some intuitive explanation.

We have replaced FEM with the familiar logistic regression models for both coevolutionary epistatic training and drug resistance inference.

Also, I feel that the results sections can benefit from some motivation, as it was not immediately clear to me why the authors were performing these analyses.

We have re-written introduction with the aim to clarify motivation, specifically, edits in the first and the last paragraphs of the introduction.
