# Supplementary material for: Forecasting drug resistant HIV protease evolution
Source: PLoS Comput Biol. 2026 Jan 27;22(1):e1013913. doi: 10.1371/journal.pcbi.1013913 (PMC12858072; doi:10.1371/journal.pcbi.1013913)
Supplement: Attachment — Submitted filename: Second_Response_to_reviewers_PLOS_HIV.docx [file pcbi.1013913.s005.docx]

Reviewer #1: This was a confusing submission to review. Effectively the entire paper has been rewritten, but some of the more important points I raised in my review haven't been addressed: isn't the state space of interactions too big for meaning fitting? why is beta interesting?

I continue to believe that there is interesting content here. But it is super hard to pull out from the manuscript. Having made some comments previously about the difficulty of reading and intepreting this manuscript, I cannot really see that things have much improved. There are 65 figure panels to digest; many of them have illegibly small text. The core ideas are very hard to connect upon reading and the mysteries above make this harder. Fig 2 and Fig 3 appear to have been swapped around. Basically -- this is a very hard paper to read. The overall message (I think) is -- we look statistically at interactions between residues and show that this motivates simulations that inform us about drug resistance. But that message gets lost at almost every stage -- it is very hard to see how the output of an individual subsection gets us towards the goal (until the end).

I suggest that the authors focus the story on Fig 3A, Fig 2A, Fig 5*, Fig 6, Fig 7*, Fig 9*, where the * means to focus on one particular drug as the lead message then illustrate variability around that. Figs 1 and 4 provide nothing to the narrative (and unless I am misunderstanding Fig 4 and Sec 2.5 are not very interesting anyway); Fig 8 is less interesting than its neighbours, and the complexity of the article needs reducing. Other content could go to the SI. The methods need to come before the results.

We have removed multiple figures and merged multiple results sections with methods to focus on the main results of this work. We moved the Methods section before the Results section, which has improved understandability of the terminology. We have reformatted figures for improved clarity.

Figures 4A and B now show plots for only one treatment regimen. Figure 4C shows plots for all treatment regimens, but we categorized them as mono-PI treatment and multi-PI treatments instead of different colors for each regimen.

Sections 2.5 and 2.6 are removed from the results to keep focus on the main story and beta is explained in Methods now.

l12 "are the least drug resistant" -- but you're talking about regimens here, right? "produce the least drug resistance"?

Edited to “...is the multi-PI treatment regimen least likely to induce drug resistance.”

l65 -- "coevolutionary information" could do with a definition here

We have added “Coevolutionary information refers to the signal of correlated mutations between amino acid residues that maintains or refines functionality and/or structure of the protein.”

l70 -- the point of simulating these trajectories isn't immediately apparent here

We have edited it as “We use these probabilities to stochastically simulate evolutionary trajectories to subsequently study statistics of emergence of drug-resistant genotypes in different treatment regimens.”

We have also edited rest of this paragraph outlining the work, aiming for better clarity.

First of all, it is essential that the methods section comes before the results section. The results section assumes a lot of knowledge about the simulations, labelling, etc that is only mentioned in the methods (there aren't even any "see Methods") links.

We have moved methods before the results.

l88 -- isn't this an impossibly large parameter space?
The authors addressed my previous point about FEM -- but not this one (which is arguably more important). Isn't the space of this set of interactions completely huge, and much too large to be spanned by a training dataset? In fig 3 top panels for example, it looks like there must be thousands (or more) of pairs of amino acids. Aren't we dramatically overfitting?
l91 -- are the observed sequences you're comparing part of the training data, or independent? If training, see overfitting question.

We use L2 regularization which is a well-established technique in the vast literature on optimized regression on overparameterized models. L2 regularization, or ridge regression, alleviates overfitting by penalizing large weights/interaction terms of logistic regression during training. It is provided in Python-sklearn’s LogisticRegression as a setting. We mention this in the Methods now.

l97 an improvement of 5% to 10% is a doubling of performance; 36% to 47% is only increasing by a third. Why "only" on l97?

We meant to say that the improvements in the minimum distances vary between 36% and 47% for the different treatment regimens, whereas the improvements in the median distances vary between 5% and 10% for the different treatment regimens. So, the values of improvements in median distances are smaller than the values of improvements in the mean distances.

We have removed this comparison between minimum and median distances altogether to keep focus on the main story.

l102 -- spatial distance ... in the sequence -- presumably this means in the folded form of the protein? As the distances between a.a. labels in the figure (which is Fig 3, not Fig 2) don't link to the distance axis.

Edited to “…spatial distance between the corresponding position in the folded protein structure.”

Corrected swapped figures.

l105 -- spatial[ly] closer

Corrected.

l106 -- the boxplots are different -- what boxplots? how does this link to the narrative in the text?

The boxplots are in the bottom panel of the (corrected) Figure 2.

We have edited the text to clarify that the boxplots are the distributions of the spatial distances in the folded structure, categorized by top 1% interacting positions vs. the rest.

This is to add evidence to the validity of epistatic interactions inferred by the logistic regression model.

l129-130 -- consistent with -- in what sense? Drug resistance and mutation propensity are not the same, not even necessarily indirectly. This needs unpacking more.

Our results showed that rare events led to a higher emergence of drug resistance and a high mutation propensity correlates with rare events. However, this needs further analysis which is out of scope of this work.

We have now removed this from the manuscript to not distract from the main story.

l125, 132 -- Fig 3 -> Fig 2

Corrected swapped figures.

Sec 2.5 -- as before, it seems that the result here is that higher temperature gives a more random search and lower temperatures give more canalised dynamics. The authors replied to my previous point about the biological relevance of beta by reiterating its algorithmic relevance as a noise parameter (inverse temperature). But without a connection to biology, why are the results obtained as a function of beta interesting? More random searches will always explore more space; less random searches will always be more constrained. Perhaps I am missing something here but I can't see what is unexpected either from a biological perspective ("more mutations explore more mutational space") or a physical ("more entropy explores more state space") picture? Unless I am missing something, this section and Fig 4 can basically be removed

We have removed section 2.5 and Figure 4 and explain inverse scaling using beta in the Methods now.

We added Section 2.4 in Methods to explain the use of beta.

Briefly -- The number of unique drug-resistant genotypes is very few at beta=1 which can skew the statistics of drug-resistant genotypes that we want to compare for different treatment regimens in subsequent analyses. Therefore, we simulate trajectories at multiple lower values of beta to explore more state space and get better estimates of the statistics of drug-resistant genotypes. This can be considered as Bayesian averaging, which is an established technique to prevent small amounts of data from skewing the estimate. It is impossible to explore the entire state space in the stochastic simulation-based framework, and this is the best that we could do currently. We ensure that for a fair comparison, the same set of beta values is used for all cases (treatment regimens).

Isn't Sec 2.6 mostly a methods section?

Section 2.6 showed that rare mutations led to emergence of a higher number of drug-resistant genotypes. We have now removed it so that it doesn’t distract from the main story.

The story in Secs 2.7 and 2.8 is interesting, as before (2.8 needs a better title).
We have edited the title (now Section 3.4) to “Mutations critical for resistance to different PIs in different treatment regimens are revealed”

l278 no verb in sentence

Edited sentence.

l278, 287, 315 -- can you give permanent DOIs rather than institutional URLs?

We cited the database in section 2.1 as directed by the official guidelines at https://hivdb.stanford.edu/pages/citation.html.

l288 -- you gave most of these abbreviations in the previous paragraph -- just give the full set there and avoid repeating?

Removed repeated full-forms.

l304 citation for sklearn?

Added citation.

l312,140 is the beta superscript a power? it's ambiguous especially given the F subscript -- it would be clearer to put the probability in brackets and raise the whole thing to the power beta
Yes, we have now raised beta as power to bracketed-probability for clarity.

Figure comments:

Why do we need figure 1? There are 22 panels and I cannot see that they provide more information than the distance statistics that are in the text.

We have removed Figure 1.

Why do we need Fig 4? More noise makes search more exploratory? As in my comment above I am not sure this message needs a figure (or mentioning at all)

We have removed Figure 4 from the main text and provide it as a supplementary figure.

Consider including summary sentences describing the science, not the detail, for each figure, e.g.
Fig 2 -- if two residues have a high inferred interaction parameter, they are likely to be close in 3D space.
Fig 3 -- the more drug treatments an isolate is exposed to, the lower mutational variability we see at the various loci under study (lower mutation propensity)

Each figure caption starts with a summary sentence now.

Fig 1 -- all text is illegibly small

Removed Fig1.

Fig 2 -- caption describes scatter plots, but these are boxplors. There isn't physical distance in this plot, which is part of the key message that this figure is supposed to show.

Corrected swapped figures 2 and 3, which was leading to mismatched captions.

Fig 3 --
- labels (points and axes) in top panels are illegibly small
- the "None" p-value in the bottom panel surely can't be zero?

Top panel: Increased font size and removed annotations to remove clutter.

Auto-formatting of p-value to print it on the plot made it 0. We have removed printing p-values and indicate significance on the plot based on p-value < 0.001 using the conventional *** notation.

Figs 5, 7, 8 -- legend text illegibly small -- why not have a single caption for all frames and make it bigger? 8's axis text is also illegible

We have updated the figures for readability and combined axes labels.

Fig 6 -- (B) bottom right point labels are illegibly small (and presumably not super interesting at such high precision)

We have removed the point labels.
