# Supplementary material for: Forecasting drug resistant HIV protease evolution
Source: PLoS Comput Biol. 2026 Jan 27;22(1):e1013913. doi: 10.1371/journal.pcbi.1013913 (PMC12858072; doi:10.1371/journal.pcbi.1013913)
Supplement: Attachment — Submitted filename: PLOS_HIV_third_response.docx [file pcbi.1013913.s006.docx]

l110 -- the "~ i" (not i) notation needs introducing here

Updated the section to clarify the notation.

l113 "overfitting" -- it would really help statistical intuition here to have: (a) the number of parameters in the base-rate-only model before any pruning; (b) the number of parameters in the interactions model before any pruning; (c) the impact of the regularisation (e.g. a distribution of the absolute parameter weights, which corresponds to but is not visible from a projection onto the x-axis in Fig 1A and 1B); (d) the number of datapoints.

Added supplementary information for each as follows.

(a) and (b) Added S2 Table. The independent models define P(sigma_i = alpha | not i) as P(sigma_i = alpha) = (number of seqs with alpha at position i)/(number of seqs). So, we report the length of the onehot encoded vectors for all treatment regimens. For epistatic models, cumulative count is shown for all positions.

(c) Added S1 Figure to show the distributions of magnitudes of all parameters (cumulative over all positions) for each treatment regimen. The title of each panel shows the number of parameters.

(d) Added S1 Table to show the number of filtered unique genotypes from the Stanford HIV database which were used to train the coevolutionary models for each treatment regimen.

l138-139 -- this is a bit of an orphan sentence and can be removed or folded in to the start of 3.1.1

Removed.

l169 "unseen" and Table 3 caption "test" -- somewhere we need the details of the training/test split. How much of the data was used for each, were the sets independent or subsets of the same data, etc.

The set of genotypes was split 80:20 into train:test (balanced labels, separately for each PI). We mention this now in Section 3.2.

Figs 5 and 6 captions -- make sure the reader has all the necessary information to understand these plots from the caption alone. e.g. describe tau in both, describe phi(tau) in Fig 6

Updated captions of Figures 5 and 6 to describe tau, phi(tau), and fractional change in phi(tau).

Updated Figure 6 caption further to explain the legends and to highlight that our analysis shows that the consensus 71A is resistance suppressing and the mutant 71V is resistance inducing.
